# Outcome after tantalum rod implantation for treatment of femoral head osteonecrosis: 26 hips followed for an average of 3 years

**DOI:** 10.1080/17453670902804877

**Published:** 2009-02-01

**Authors:** Sokratis E Varitimidis, Apostolos P Dimitroulias, Theophilos S Karachalios, Zoe H Dailiana, Konstantinos N Malizos

**Affiliations:** Department of Orthopedic Surgery, School of Medicine, University of ThessaliaLarissaGreece

## Abstract

**Background and purpose** Tantalum rod implantation has recently been proposed for treatment of early stages of femoral head osteonecrosis. The purpose of our study was to report the early results of its use in pre- and post-collapse stages of the disease.

**Methods** We studied prospectively 27 patients who underwent tantalum rod implantation for treatment of nontraumatic femoral head osteonecrosis between December 2000 and September 2005. Patients were evaluated radiologically and clinically using the Steinberg classification and the Harris hip score (HHS). Disease stage varied between stages II and IV. Mean follow-up time was 38 (15–71) months.

**Results** 1 patient (1 hip) died 15 months after surgery for reasons unrelated to it. 13 of 26 hips remained at the same radiographic stage, and 13 deteriorated. Mean HHS improved from 49 to 85. 6 patients required conversion to total hip arthroplasty. When the procedure was used for stages III and IV, both radiological outcome and revision rates were worse than for the stage II hips. There was, however, no difference in postoperative HHS between patients at pre- and post-collapse stages at the time of initial evaluation. Survivorship, with revision to THA as the endpoint, was 70% at 6 years.

**Interpretation** The disease process does not appear to be interrupted, but there was a significant improvement in hip function initially in most hips. Tantalum rod implantation is a safe “buy-time” technique, especially when other joint salvage procedures are not an option. Appropriate patient selection and careful rod insertion are needed for favorable results.

## Introduction

The femoral head is the most vulnerable site for development of osteonecrosis. The incidence is increasing: every year 10,000–20,000 new cases are diagnosed in the USA and 5–12% of total hip arthroplasties performed each year are due to osteonecrosis (Mont and Hungerford 1995). Although early diagnosis has been facilitated by the use of bone scan and MRI, there is no universally accepted treatment—especially at the early stage of disease.

The joint-preserving procedures include core decompression with or without bone grafting (Camp and Colwell 1986, Steinberg et al. 2001), rotational osteotomy (Sugioka et al. 1982), varus or valgus osteotomy (Dresher et al. 2003), nonvascularized fibula grafting (Plakseychuk et al. 2003), vascularized fibula grafting (Malizos et al. 1995, Urbaniak et al. 1995, Sotereanos et al. 1997, Plakseychuk et al. 2001), and—recently—implantation of trabecular metal tantalum rod with 3 reports (Tsao et al. 2005, Veillette et al. 2006, Shuler et al. 2007). We evaluated the clinical and radiological outcome following implantation of a porous tantalum implant for treatment of pre- and post-collapse stages of nontraumatic femoral head osteonecrosis.

## Patients and methods

We studied prospectively 27 patients (32 hips) who underwent tantalum rod implantation for treatment of nontraumatic femoral head osteonecrosis between December 2000 and September 2005. Although the preferred joint-preserving procedure at our institution is vascularized fibular graft, we proposed tantalum rod to patients who were medically unfit or unwilling to undergo this longer procedure with long rehabilitation, or to young patients with post-collapse disease who were reluctant to have a total hip replacement. The study was approved by the ethics committee of our institution (date of issue 1/11/2000, registration no. 035) and all patients gave their informed consent.

The study included 23 male and 4 female patients with a median age of 36 (15–55) years. 1 patient (1 hip) died 15 months postoperatively for reasons unrelated to surgery (sickle cell crisis), and was excluded from the study. Osteonecrosis was idiopathic in 8 patients, secondary to cortisone intake in 15, due to alcohol intake in 2, and secondary to sickle cell anemia in 1 patient. None of the patients had any prior treatment for the osteonecrosis. The preoperative stage was stage II in 9 patients, stage III in 7, and stage IV in 10 patients. Mean follow-up was 38 (15–71) months. No patients were lost to follow-up.

5 patients had bilateral tantalum rod implantation. For these 5 patients, 1 of the hips was selected using a computer-generated random sequence in order to fulfill the requirement of data independence for statistical analysis purposes.

Patients were evaluated preoperatively and at the end of the follow-up period, both clinically and radiologically using the Harris hip score (HHS) and Steinberg classification (Harris 1969, Steinberg et al. 1995). The mean preoperative HHS was 49 (SD 19). All patients underwent preoperative radiography and MR imaging of the hips. Postoperative Steinberg stage evaluation was based on plain radiographs.

Two independent observers other than the surgeons did the radiographic evaluation. In cases of discordance, a unanimous final judgment was made. We made no attempt to examine the intraobserver or interobserver variability. Throughout the study, clinical evaluation was done by a single observer (not a surgeon).

Finally, an MRI-based volumetric method was used to quantify the size of the lesion on the preoperative MRI scans. We used a semi-automated technique in which the area with abnormal signal was outlined for each MRI slice using the tracing tool of the ANALYZE image analysis software, version 8.1 (Mayo Foundation, Rochester, MN). We have reported details of the technique elsewhere (Malizos et al. 2001).

The implant used was a porous tantalum rod (10 mm in diameter) (Trabecular Metal; Zimmer Trabecular Metal Technology, Allendale, NJ). It was inserted through a small lateral incision under biplanar fluoroscopic guidance, and it was aimed at the osteonecrotic lesion to support the subchondral bone. The tip of the rod was advanced not more than 5 mm from the articular surface. We did not place any bone graft in the femoral head. Although according to the technique originally described the lateral part of the rod should abut the lateral femoral cortex, we used shorter rods (either 70 or 75 mm) so that the rod ended distally at the cancellous bone of the trochanteric area. With this method, if a later arthroplasty is required, the initial cortical defect will have been filled with bone. The distal threads and the high frictional stability of the implant prevent backing out.

There were no perioperative complications. Postoperative rehabilitation included partial weight bearing for 6 weeks.

### Statistics

We used the Wilcoxon signed ranks test to compare the differences in pre- and postoperative Steinberg classification and HHS. The Wilcoxon-Mann-Whitney test was used to compare postoperative Steinberg and HHS scores between primary and secondary etiology groups. 95% bootstrap confidence intervals (CIs) for differences in median values follow p-values were calculated using the Wilcoxon-Mann-Whitney test. Spearman’s rho (ρ) was calculated for the assessment of the correlation of age to postoperative Steinberg and HHS scores and to assess the correlation between lesion size and outcome. The endpoint for the survival analysis was hip arthroplasty and the results were presented with a Kaplan-Meier survivorship curve. The standard error of the cumulative survival was calculated using Greenwood’s formula. All reported p-values are two-tailed with p < 0.05 being considered significant. Analyses were conducted using SPSS version 15.0 (SPSS Inc., Chicago, IL) and S-plus version 8.0 (Insightful Corp., Seattle, WA).

**Figure 1.**
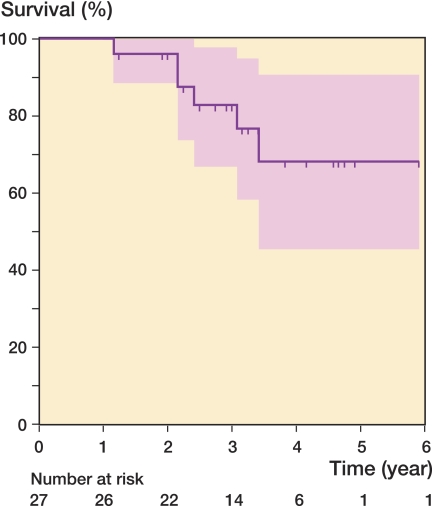
Kaplan-Meier survivorship curve with 95% pointwise confidence intervals using total hip arthroplasty as the endpoint. Vertical marks indicate censored data.

## Results

**Figure 2.**
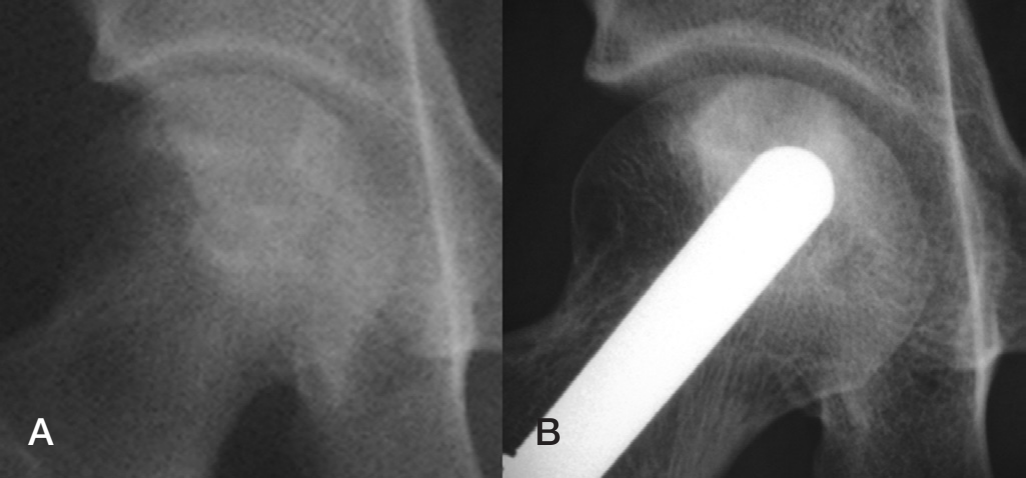
Right hip of a 15-year-old girl with osteonecrosis of the femoral head secondary to cortisone intake for marrow transplantation. A. Preoperatively, Steinberg stage II with HHS of 85. B. 5 years later, radiographic Steinberg staging remained unchanged and HHS was 100.

13 of the 26 hips did not have any radiographic progression of the disease at the end of the follow-up. The preoperative Steinberg staging of these 13 hips was: 5 stage II (Figure 2), 2 stage III, and 6 stage IV. Of the 13 hips with radiographic deterioration, 4 were stage II, 5 were stage III, and 4 were stage IV preoperatively.

**Figure 3.**
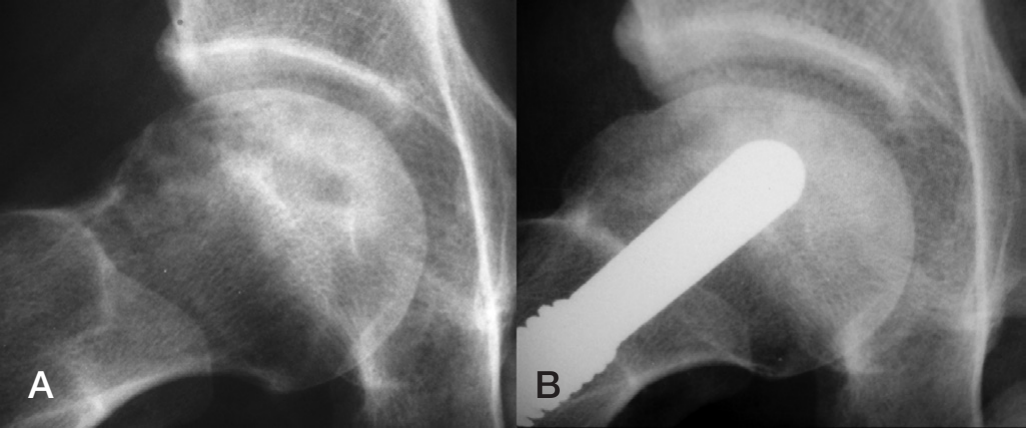
Right hip of a 38-year-old man with idiopathic osteonecrosis. A. Preoperatively, Steinberg stage IV with HHS of 63. B. 3.5 years later, Steinberg stage remained unchanged and HHS was 93.

There was better radiological outcome for patients at precollapse stage at the time of operation (4 of 9 stage II hips deteriorated) when compared to those at post-collapse stages (9 of 17 stage III and IV hips deteriorated) (p = 0.02). Interestingly, there was no statistically significant difference in postoperative HHS between these two groups of patients (p = 0.7, 95% bootstrap CI for median differences (–23, 19)). Moreover, HHS at last follow-up was not statistically significantly greater (mean HHS 90, SD 13) in patients with unchanged Steinberg stage, when compared to those with radiographic progression (mean HHS 79, SD 15) (p = 0.1, 95% bootstrap CI for median differences (–2, 33)) (Figure 3).

There were no significant differences in the radiological outcome (p = 0.3, 95% bootstrap CI for median differences (–2, 1)) and the clinical outcome (p = 1.0, 95% bootstrap CI for median differences (–20, 19.5)) between the subgroup of patients with a serious underlying disease (e.g. blood malignancy, lupus erythematosus, transplant patients) and those without. Furthermore, we could not find any significant difference in the postoperative Steinberg stage (p = 1.0, 95% bootstrap CI for median differences (–1, 1)) and HHS (p = 0.4, 95% bootstrap CI for median differences (–31, 17)) when we compared the primary and the secondary etiology groups of patients.

There was no significant correlation between age and postoperative Steinberg (correlation coefficient, r = 0.17) or HHS score (r = –0.15).

It is notable that when all hips were grouped together, despite radiological deterioration over time (p < 0.001, 95% bootstrap CI for median differences (–2, –1)) there was a clinical improvement compared to the preoperative status of the patients (p < 0.001, 95% bootstrap CI for median differences (24, 52)). The mean HHS increased from 49 (SD 19) preoperatively to 85 (SD 15) postoperatively. The mean estimated survival time of the implant was 59 months (SE 4 months, 95% CI: 50–67 months). There were no cases of tantalum rod breakage, loosening, or subsidence.

We found a statistically significant positive correlation between the preoperative volume of the osteonecrotic lesion and the postoperative radiographic stage of the disease (ρ = 0.73). This correlation was noted not only in hips that had been treated prior to femoral head collapse (ρ = 0.89) but also in the post-collapse group of hips (ρ = 0.66). There was no correlation between the preoperative volume of necrotic femoral head and postoperative HHS (ρ = –0.41).

**Figure 4.**
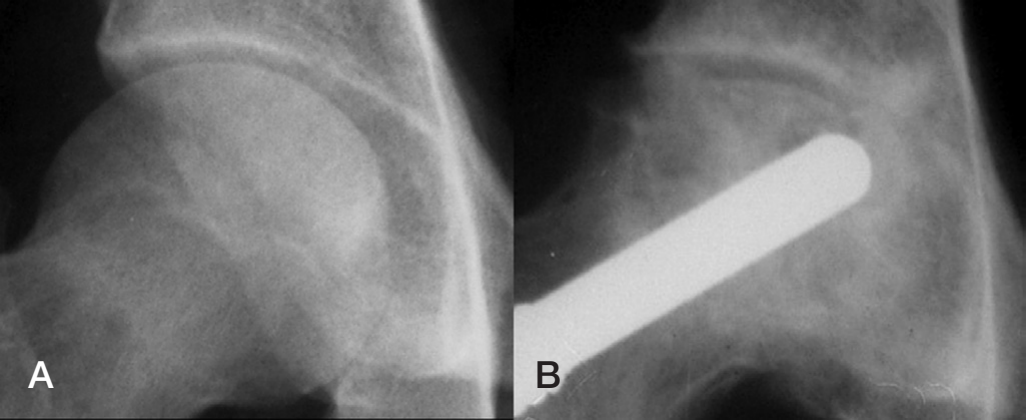
27-year-old man with osteonecrosis secondary to cortisone intake for Hodgkin lymphoma. A. Preoperatively, the disease was clas-sified as Steinberg stage II. B. 3 years later, the hip had deteriorated radiographically to Steinberg stage VI and required revision to THA.

During the study period, 6 of the 26 hips underwent revision to a total hip arthroplasty at an average of 29 months (range 14–41 months, SD 9.2). Only 1 of the 6 hips was stage II at the time of the index operation (Figure 4); the others were stage III and IV. Overall survival was 70% (CI: 52–95) at 71 months. All revisions were done for pain relief. There were no intraoperative complications during the revision procedure. The tantalum implant was removed uneventfully in all 6 hips: the cortex of the femoral neck was cut circumferentially at the desired level with a reciprocating saw until the rod was encountered. The head was then removed in a piecemeal fashion by performing osteotomies at a plane tangential to the rod. Multiple drill holes were then made at the circumference of the rod, and finally the implant was removed using some back-and-forth movements. The additional operative time that was required was 5–10 min. In all 6 patients, an uncemented stem was implanted without any intraoperative complications. This method did not render the femoral neck vulnerable to fracture. We believe that even with cemented prostheses, removal of the rod would not be troublesome as the entrance point of the rod would have healed adequately enough in order to prevent outflow of the cement.

## Discussion

Preservation of an osteonecrotic femoral head depends on prevention of collapse of the structurally compromised necrotic bone. Non-surgical management with partial weight bearing can only be selected for early stages and very small lesions. Even in such cases, it has been proven to be ineffective in 80–90% of patients (Mont et al. 1996, Hungerford and Mont 1998). Other nonoperative treatment options include hyperbaric oxygen (Reis et al. 2003), administration of biphosphonates (Bowers et al. 2004, Lai et al. 2005), lipid-lowering agents (Pritchett 2001), prostaglandin analogs (Disch et al. 2005), low-molecular-weight heparin (Glueck et al. 2005), and high-energy shock waves (Ludwig et al. 2001).

A meta-analysis of studies with core decompression or nonoperative treatment (Castro and Barrack 2000) demonstrated success rates (defined as no further surgical intervention) of 61%, 59%, and 25% for nonoperative treatment of osteonecrosis of Steinberg stages I, II and III, respectively. Core decompression, one of the least invasive surgical procedures, may be efficacious in early stages and small-size lesions of osteonecrosis, although concerns have been raised because of its potential to weaken the cancellous bone within and adjacent to the necrotic area (Scully et al. 1998, Lavernia and Sierra 2000). Finite-element models have demonstrated that core drilling of a lesion could have major implications for the structural integrity of the head (Brown et al. 1993). On the other hand, core decompression that was originally performed as a diagnostic procedure has been advocated as a technique capable of interrupting the disease process when performed before subchondral collapse. A meta-analysis that evaluated studies with core decompression technique or nonoperative treatment for femoral head osteonecrosis (Castro and Barrack 2000) found that further surgical intervention was necessary in 16%, 37%, and 71% of cases after core decompression of osteonecrosis at Steinberg stages I, II, and III, respectively.

Radiographic progression after core decompression has been well documented in the literature. In a 2–6-year follow-up study, Smith et al. (1995) found a rate of 41%, 66%, 96%, and 100% of radiographic deterioration after core decompression for Steinberg stages I, II, III, and IV, respectively. Similarly, Fairbank et al. (1995) reported rates of 16%, 58%, and 90% radiographic progression for Steinberg stages I, II, and III–IV, respectively. Two other studies found, in the short (early) term, a radiographic progression after core decompression in three-quarters of cases (Stulberg et al. 1991, Koo et al. 1995). The best results with this procedure can be expected with segmental lesions (intact lateral pillar), prior to collapse and with radiographic signs of sclerotic bone (Mont et al. 1998). In our series radiographic deterioration was observed in 4 of 9 stage II hips and in 9 of 17 stage III and IV hips, which compares favorably with the results of the above studies. This suggests that the benefit obtained after tantalum rod implantation cannot be attributed solely to the core decompression effect.

Cortical bone grafting of the femoral head may offer structural reinforcement, particularly if the graft penetrates deeply into the superior-central or lateral aspect of the lesion (Penix et al. 1983). Supporting the articular surface with nonvascularized fibula grafting has not provided satisfactory long-term results. In a comparative study, Plakseychuk et al. (2003) reported only one-third good and excellent results with the use of nonvascularized fibula whereas they reported two-thirds good and excellent results with a vascularized fibula graft.

Even with this demanding technique, outcomes seem to be compromised with time, leading to a hip arthroplasty in many patients (Malizos et al. 1995, Xenakis et al. 1997, Malizos et al. 2004, Shuler et al. 2007).

Recently, trabecular metal rods implanted in the femoral head with or without the addition of autologous growth factors have been advocated as an alternative to prevent collapse in stages I–III. Because of the surface characteristics of these metal implants (pore dimensions and 3D structure similar to the cellular structure of bone), high-volume bone ingrowth is encouraged and collapse is prevented. The porous tantalum is a highly biocompatible material with a porosity of 75% and pore size of 430 µm. Pores are fully interconnected and the microtexture of the material (dodecahedron) permits rapid bony ingrowth. Elastic modulus falls between that of cortical and subchondral bone and compressive strength is between that of cortical and cancellus bone. Initial stability at implantation is high due to its high coefficient of friction.

The use of tantalum rods for the treatment of femoral head osteonecrosis was first proposed in 1997 (Pedersen et al. 1997). The investigators used an MR-based 3D finite element model of femoral head osteonecrosis to study the mechanical effects of a porous tantalum rod in the necrotic femoral head, and they concluded that the porous tantalum rod constitutes a reasonable mechanical substitute for a fibular graft as it effectively reduces peak stress-strain ratio. Heiner et al. (1998) found that mean flexural stiffness was 12 Nm^2^ for the porous tantalum, whereas the fibular graft had a flexural stiffness that ranged from 11 to 17 Nm^2^. In another laboratory study, Heiner et al. (2001) demonstrated that the porous tantalum implant can effectively support the subchondral bone of the femoral head and that the strength of the implant is more than 9 times greater than the loading stress exerted on the implant.

Since then, very few clinical studies have been published. Tsao et al. (2005) evaluated the porous tantalum implant in a multicenter study that included 113 hips in 98 patients. At 4-year follow-up, the survival rate for preoperative stage II hips was 70% (with revision to THA as endpoint) and the average HHS was 83. Best results were obtained in patients in whom steroid intake was the underlying factor for osteonecrosis. In another study (Shuler et al. 2007), tantalum rod was compared to free vascularized fibula graft for treatment of early-stage femoral head osteonecrosis (Steinberg I and II). The survival rate was 86% at a mean of 3 years, and it was superior to survival in patients treated with free vascularized fibula grafting. Information on whether or not there was radiographic progression was not given in these two studies. Veillette et al. (2006) studied retrospectively 58 hips after tantalum rod implantation. Most hips were at Steinberg stage II at diagnosis and average follow-up was 2 (0.5–4.5) years. Radiographic progression was present in 16 hips. The 4-year overall survival was 68% (with revision to THA as endpoint). In our study, we also used porous tantalum rods for patients with stage III and IV lesions—especially in patients with serious underlying medical problems. Despite that, we found survival rates (70% at 4 years) that are similar to those reported by Tsao et al. (70% at 4 years) and Veillette et al. (68% at 4 years). Radiographic progression and revision to hip arthroplasty were more common in patients at the post-collapse stage at the time of the index procedure.

Despite the radiographic deterioration that was seen in 13 of 26 hips, HHS improved. We found no significant difference in postoperative HHS between patients at pre- and post-collapse stages at the time of the initial evaluation. Moreover, postoperative HHS was similar in the group of patients with radiographically stable lesions over the course of the study and those with radiographic deterioration. There was a correlation between the volume of the lesion and the postoperative Steinberg stage, but not between volume and the final clinical score.

The frequency of radiologic progression observed in our study was less than that reported for core decompression alone. This indicates that the tantalum rod implant provides a meaningful structural support for the subchondral plate in addition to the core decompression achieved by the porous nature of the material.

We did not find any correlation between disease etiology and postoperative clinical and radiological scores (p = 0.42 and p = 0.97, respectively). This contrasts with a recent study (Veillette et al. 2006) in which higher survival rates were found in patients without chronic systemic diseases. The two studies cannot be directly compared, however, as tantalum rod was used for postcollapse stages of osteonecrosis as well.

In summary, we found that use of the porous tantalum rod is a relatively easy, minimally invasive, and reproducible procedure that can provide, at least in the short term, satisfactory functional improvement at both pre- and post-collapse stages of hip osteonecrosis. To a lesser extent, it can preserve the structural integrity of the femoral head. This is best achieved in hips at the pre-collapse stage.

It would be interesting for future studies to do randomized comparisons between the outcomes of tantalum rod implantation and core decompression alone, or with implantation of fibular strut allograft.
